# A Quantum-Mechanical Looking Behind the Scene of the Classic G·C Nucleobase Pairs Tautomerization

**DOI:** 10.3389/fchem.2020.574454

**Published:** 2020-11-26

**Authors:** Ol'ha O. Brovarets', Alona Muradova, Dmytro M. Hovorun

**Affiliations:** ^1^Department of Molecular and Quantum Biophysics, Institute of Molecular Biology and Genetics, National Academy of Sciences of Ukraine, Kyiv, Ukraine; ^2^Department of Molecular Biotechnology and Bioinformatics, Institute of High Technologies, Taras Shevchenko National University of Kyiv, Kyiv, Ukraine

**Keywords:** DNA, RNA, G•C base pair, tautomerization mechanism, wobblization, wobble base pair, Levitt base pair, Löwdin's base pair

## Abstract

For the first time, at the MP2/6-311++G(2df,pd)//B3LYP/6-311++G(d,p) level of theory, a comprehensive quantum-mechanical investigation of the physico-chemical mechanism of the tautomeric wobblization of the four biologically-important G·C nucleobase pairs by the participation of the monomers in rare, in particular mutagenic, tautomeric forms (marked with an asterisk) was provided. These novel tautomeric transformations (wobblization or shifting of the bases within the pair) are intrinsically inherent properties of the G·C nucleobase pairs. In this study, we have obtained intriguing results, lying far beyond the existing representations. Thus, it was shown that Löwdin's G^*^·C^*^(WC) base pair does not tautomerize according to the wobblization mechanism. Tautomeric wobblization of the G^*^·C^*^(rWC) (relative Gibbs free energy ΔG = 0.00/relative electronic energy ΔE = 0.00 kcal·mol^−1^) (“r”—means the configuration of the base pair in reverse position; “WC”—the classic Watson-Crick configuration) and G^*t^·C^*^(H) (ΔG = −0.19/ΔE = 0.29 kcal·mol^−1^) (“H”—Hoogsteen configuration;”t” denotes the O6H hydroxyl group in the *trans* position) base pairs are preceded by the stages of the base pairs tautomerization by the single proton transfer (SPT). It was established that the G^*t^·C^*^(rH) (ΔG = 2.21/ΔE = 2.81 kcal·mol^−1^) base pair can be wobbled through two different pathways *via* the traditional one-stage mechanism through the TSs, which are tight G^+^·C^−^ ion pairs, stabilized by the participation of only two intermolecular H-bonds. It was found out that the G·C base pair is most likely incorporated into the DNA/RNA double helix with parallel strands in the G^*^·C^*^(rWC), G·C^*^(rw_wc_), and G^*^·C(rw_wc_) (“w”—wobble configuration of the pair) tautomeric forms, which are in rapid tautomeric equilibrium with each other. It was proven that the G^*^·C^*^(rWC) nucleobase pair is also in rapid tautomeric equilibrium with the eight tautomeric forms of the so-called Levitt base pair. It was revealed that a few cases of tautomerization *via* the DPT of the nucleobase pairs by the participation of the C8H group of the guanine had occurred. The biological role of the obtained results was also made apparent.

## Introduction

Shortly after the establishment of the spatial organization of the DNA molecule by James Watson and Francis Crick (Watson and Crick, 1953a,b), the tautomeric hypothesis was formulated (Watson and Crick, 1953b; Crick and Watson, 1954), which considers the transformation or transition of the nucleotide bases from the main (canonical) into the rare (mutagenic) tautomeric form as the main source of the origin of spontaneous point mutations. Since that time, the topic of tautomerism has remained active over the decades to the present day (Löwdin, 1963, 1966; Topal and Fresco, 1976; Florian et al., 1994; Gorb et al., 2004; Brovarets' et al., 2014; Godbeer et al., 2015; Turaeva and Brown-Kennerly, 2015).

However, up until recently it was considered that only a few unusual tautomers existed for the G·C Watson-Crick nucleobase pair (Pous et al., 2008; Alvey et al., 2014; Brovarets' and Hovorun, 2014a; Nikolova et al., 2014; Poltev et al., 2016; Szabat and Kierzek, 2017; Brovarets' et al., 2019a,b; Srivastava, 2019). In particular, tautomerization *via* the double proton transfer (DPT) has been carefully investigated in the reverse Löwdin G^*^·C^*^(rWC), Hoogsteen (H) G^*^′·C^*^(H), and reverse Hoogsteen G^*^′·C^*^(rH) base pairs (Brovarets' et al., 2019b), leading to the novel structures: G·${\text{C}}_{\text{O}2}^{*}$(rWC), ${\text{G}}_{\text{N}2}^{*}\cdot$C(rWC), G^*′^_N2_·C(rWC), ${\text{G}}_{\text{N}7}^{*}\cdot$C(H), and G^*′^_N7_·C(rH).

Eventually a great contribution into the further development of the tautomeric hypothesis was made by Per-Orlov Löwdin (Löwdin, 1963, 1966) and Topal and Fresco (Topal and Fresco, 1976; Brovarets' et al., 2014). Thus, Per-Orlov Löwdin expressed the revolutionary, non-trivial opinion that the ability of the nucleotide bases to transform into the rare tautomeric form is provided by the electronic structure of the canonical DNA base pairs and qualitatively substantiated this assumption from the position of quantum mechanics. Subsequently, Topal and Fresco elaborated this approach in more detail, by using simple and visual models, and extended it for the explanation of the limited accuracy of codon-anticodon recognition (Topal and Fresco, 1976; Brovarets' et al., 2014).

In recent years, an alternative view in this area of the research was suggested, which could be characterized as the “Renaissance” of the tautomeric hypothesis [see Chapter Brovarets' and Hovorun (2018) and bibliography provided there]. According to this investigation the new, unusual pathways of the tautomeric interconversions between wobble (w) and Watson-Crick (WC) base pairs have been provided (Brovarets' and Hovorun, 2009, 2015a,b,c,d,e,f):

- for usual A·T and G·C DNA base pairs: A·T(WC)↔A^*^·T(w)/A$\cdot {\text{T}}_{\text{O}2}^{*}$(w)/A·T^*^(w) and G·C(WC)↔G·${\text{C}}_{\text{↑}}^{*}$(w)/G^*^·C_↓_(w)/G·${\text{C}}_{\text{↓}}^{*}$(w)/G^*^·C_↑_(w) (Brovarets' and Hovorun, 2015a);
- for unusual purine-pyrimidine wobble A·C and G·T DNA base pairs: A·C(w)↔A·C^*^(WC) and G·T(w)↔G^*^·T(WC) (Brovarets' and Hovorun, 2009, 2015b,c);
- for incorrect purine-purine A·A, G·G, and A·G DNA base pairs: A·A(w)↔A^*^·A(WC), G·G(w)↔G^*^·G(WC), A·G(WC)↔A·${\text{G}}_{\text{↓}}^{*}$(w), A·G(WC)↔A^*^·G_↑_(w) (Brovarets' and Hovorun, 2015d,e);
- for pyrimidine-pyrimidine base pairs: C·T(WC)↔C^*^·T_↑_(w), C·T(WC)↔C·${\text{T}}_{\text{↓}}^{*}$(w), T·T(w)↔T·T^*^(WC), C·C(w)↔C·C^*^(WC) (Brovarets' and Hovorun, 2015f).

Thus, by utilizing modern quantum-mechanical (QM) methods, the mechanisms of the mutagenic tautomerization of the pairs of nucleotide bases were investigated in detail, which were revealed to be active players in the field of spontaneous point mutagenesis (Brovarets' and Hovorun, 2018). It was established, in which cases Löwdin's approach was adequate and in which cases another approach should be reconsidered and supplemented.

Thus, it was suggested that the mechanism of the mutagenic tautomerization of the DNA base pairs, in particular classic Watson-Crick pairs, are accompanied by the mutual shifting (wobblization) of the bases one relative to the other into the minor or major DNA grooves at the intrapair sequential proton transfer (Brovarets' and Hovorun, 2015a; Brovarets' et al., 2019a). This valuable finding enables researchers to figure out, how the incorrect DNA base pairs, which architecture is different from the Watson-Crick configuration, can acquire the enzymatically-competent conformation, that guarantees their successful chemical incorporation into the composition of the main carrier of the genetic information—DNA—by the high-fidelity DNA-polymerase. Notably, even though these theoretical approaches have been realized in quite basic model objects, they correctly reflect the real state-of-affairs at the macromolecular level, since they have been experimentally confirmed for macromolecular objects.

In this research, the objects of the investigation have been extended—except the Watson-Crick (WC) nucleobase pair, to the other biologically-important G·C nucleobase pairs—reverse Watson-Crick G·C(rWC), Hoogsteen G·C(H), and reverse Hoogsteen G·C(rH). Also, it was exactly established why the classic A·T(WC) DNA base pair was selected for the construction of the genetic material (Brovarets' and Hovorun, 2009, 2015a,b,c,d,e,f; Brovarets' et al., 2018a). The novel mechanism of the mutagenic tautomerization of the biologically-important A·T DNA base pairs through the quasi-orthogonal transition state and also through the protonated amino-group (Brovarets' et al., 2018b,c,d,e,f) was revealed for the first time. Based on these data an assumption was expressed about their possible biological role.

At the same time, investigations into the mechanisms of the mutagenic tautomerization of the pairs of nucleotide bases seemed to be quite a complicated issue, which may not be evident at a first glance. Thus, recent investigations into the tautomerization mechanisms of the biologically-important G·C nucleobase pairs, in which monomers are in the rare, in particular mutagenic, tautomeric form, continue to challenge researchers by its mystery (Brovarets' et al., 2019a,b).

It is still not possible to formulate simple physico-chemical rules, that would predict the course of these biologically important processes. Obviously, this is due to the fact that despite the enormous theoretical and experimental efforts of researchers, the present material remains insufficient for its final generalization.

This work aims to deepen the existing ideas about the microstructural mechanisms of the tautomerization of the biologically important pairs of nucleobases using the example of the G·C base pair (Brovarets' and Hovorun, 2014a), for which both monomers are in the rare tautomeric form.

Such a task is completely substantiated—we have investigated a few surprising tautomerizations, which significantly expand the existing ideas on tautomerization mechanisms and their biological applications. They will be outlined and discussed in more detail below.

## Computational Methods

### Density Functional Theory Calculations of the Geometry and Vibrational Frequencies

Equilibrium geometries of the investigated nucleobase pairs and the transition states (TSs) of their mutual tautomeric transformations, as well as their harmonic vibrational frequencies have been calculated at the B3LYP/6-311++G(d,p) level of QM theory (Hariharan and Pople, 1973; Krishnan et al., 1980; Lee et al., 1988; Parr and Yang, 1989; Tirado-Rives and Jorgensen, 2008), using the Gaussian'09 program package (Frisch et al., 2010). An applied level of theory has proved itself to be successful for the calculations of similar systems (Brovarets' and Hovorun, 2010a,b, 2015g; Matta, 2010; Brovarets' et al., 2015). A scaling factor that is equal to 0.9668 has been applied in the present work for the correction of the harmonic frequencies of all complexes and TSs of their tautomeric transitions (Palafox, 2014; Brovarets' and Hovorun, 2015g; Brovarets' et al., 2015; El-Sayed et al., 2015). We have confirmed the local minima and TSs, localized by a synchronous transit-guided quasi-Newton method (Peng et al., 1996), on the potential energy landscape by the absence or presence, respectively, of the imaginary frequency in the vibrational spectra of the complexes. We applied standard TS theory for the estimation of the activation barriers of the tautomerization reaction (Atkins, 1998).

All calculations have been carried in the continuum with ε = 1, that adequately reflects the processes occurring in real biological systems without deprivation of the structurally functional properties of the bases in the composition of DNA/RNA and satisfactorily models the substantially hydrophobic recognition pocket of the DNA-polymerase machinery as a part of the replisome (Bayley, 1951; Dewar and Storch, 1985; Petrushka et al., 1986; García-Moreno et al., 1997; Mertz and Krishtalik, 2000; Brovarets' and Hovorun, 2014a,b).

### Single Point Energy Calculations

We continued geometry optimizations with electronic energy calculations as single point calculations at the MP2/6-311++G(2df,pd) level of theory (Frisch et al., 1990; Kendall et al., 1992).

The Gibbs free energy G for all structures was obtained in the following way:

(1) $$\begin{array}{r} \text{G}={\text{E}}_{\text{el}}+{\text{E}}_{\text{corr}}, \end{array}$$

where E_el_–electronic energy, while E_corr_–thermal correction.

### Evaluation of the Interaction Energies

Electronic interaction energies ΔE_int_ have been calculated at the MP2/6-311++G(2df,pd) level of theory as the difference between the total energy of the base pair and energies of the monomers, which have been corrected for the basis set superposition error (BSSE) (Boys and Bernardi, 1970; Gutowski et al., 1986) through the counterpoise procedure (Sordo et al., 1988; Sordo, 2001).

### QTAIM Analysis

Bader's quantum theory of atoms in molecules (QTAIM) (Bader, 1990; Matta and Hernández-Trujillo, 2003; Matta et al., 2006; Cukrowski and Matta, 2010; Keith, 2010; Matta, 2014; Lecomte et al., 2015), using the program package AIMAll (Keith, 2010), was applied to analyze the electron density distribution. The presence of the bond critical point (BCP), namely the so-called (3,-1) BCP, and a bond path between the hydrogen donor and acceptor, as well as the positive value of the Laplacian at this BCP (Δρ > 0), were considered as criteria for the H-bond formation (Bader, 1990; Matta and Hernández-Trujillo, 2003; Matta et al., 2006; Cukrowski and Matta, 2010; Matta, 2014; Lecomte et al., 2015). Wave functions were obtained at the level of QM theory used for geometry optimization.

The atomic numbering scheme used for the nucleobases is conventional (Saenger, 1984). In this study mutagenic or rare tautomeric forms of the bases (Brovarets' and Hovorun, 2009, 2014a, 2015a,b,c,d,e,f; Brovarets' and Hovorun, 2018; Brovarets' et al., 2018a,b,c,d,e,f; Brovarets' et al., 2019a,b) are denoted by the asterisk.

## Obtained Results and Their Discussion

**Figure 1:**
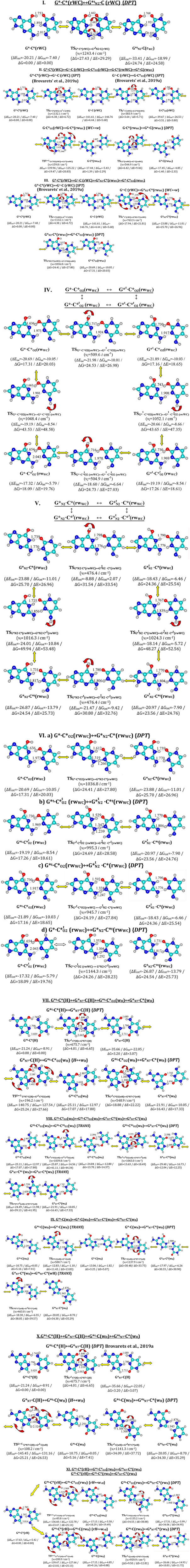
Investigated pathways of the tautomeric wobblization of the biologically-important G·C nucleobase pairs – G*·C*(rWC), G*·C*(H) and G*·C*(rH) pairs obtained at the MP2/6-311++G(2df,pd)//B3LYP/6-311++G(d,p) level of QM theory. ΔG-relative Gibbs free energy and ΔE-electronic energy (in kcal·mol^−1^; MP2/6-311++G(2df,pd)//B3LYP/6-311++G(d,p) level of QM theory); ΔE_int_-electronic and ΔG_int_-Gibbs free energies of the interaction (MP2/6-311++G(2df,pd)//B3LYP/6-311++G(d,p) level of QM theory, in kcal·mol^−1^). ν_i_–imaginary frequency. Intermolecular AH…B H-bonds are designated by dotted lines, their lengths H…B are presented in angstroms.

**Figure 2:**
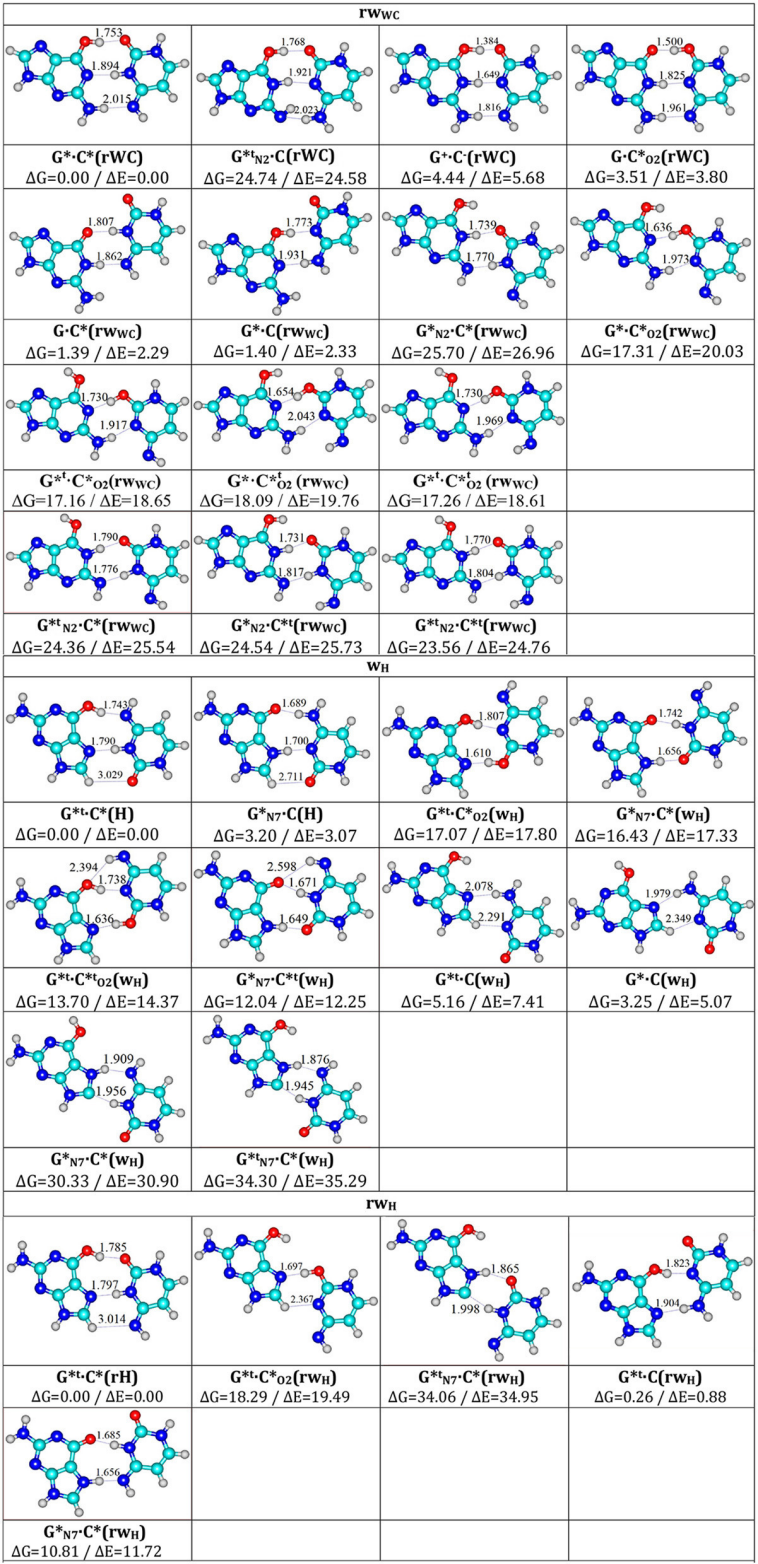
Total geometries of the investigated G·C nucleobase pairs, corresponding to the local minima, presented altogether with their relative Gibbs free energies ΔG and electronic energies (ΔE in kcal·mol^−1^ under normal conditions) obtained at the MP2/6-311++G(2df,pd)//B3LYP/6-311++G(d,p) level of QM theory.

So, based on the obtained data, let us firstly formulate the basic results, which have been obtained for the first time and which have the closest connection to the structural biology and molecular biophysics (Figures 1, 2, Table 1).

**Table 1:** Energetic characteristics of the tautomers of the G·C nucleobase pairs obtained at the MP2/6-311++G(2df,pd)//B3LYP/6-311++G(d,p) level of QM theory in vacuum (ε = 1) (see Figure 2).

**N**	**G·C complex**	**ΔG^a^**	**ΔE^b^**
**rWC/rw**_**WC**_			
1	G*·C*(rWC)	0.00	0.00
2	G·C*(rw_WC_)	1.39	2.29
3	G*·C(rw_WC_)	1.40	2.33
4	G·C*_O2_(rWC)	3.51	3.80
5	G^+^·C^−^(rWC)	4.44	5.68
6	G^*t^·C*_O2_(rw_WC_)	17.16	18.65
7	G^*t^·${\text{C}}_{\text{O}2}^{*\text{t}}$(rw_WC_)	17.26	18.61
8	G*·C*_O2_(rw_WC_)	17.31	20.03
9	G*·${\text{C}}_{\text{O}2}^{*\text{t}}$(rw_WC_)	18.09	19.76
10	${\text{C}}_{\text{N}2}^{*\text{t}}$·C*(rw_WC_)	24.36	25.54
11	G*_N2_·C^*t^(rw_WC_)	24.54	25.73
12	${\text{C}}_{\text{N}2}^{*\text{t}}$·C^*t^(rw_WC_)	23.56	24.76
13	${\text{C}}_{\text{N}2}^{*\text{t}}$·C(rWC)	24.74	24.58
14	G*_N2_·C*(rw_WC_)	25.70	26.96
**H/w**_**H**_			
15	G^*t^·C*(H)	0.00	0.00
16	G*_N7_·C(H)	3.20	3.07
17	G*·C(w_H_)	3.25	5.07
18	G^*t^·C(w_H_)	5.16	7.41
19	G*_N7_·C^*t^(w_H_)	12.04	12.25
20	G^*t^·${\text{C}}_{\text{O}2}^{*\text{t}}$(w_H_)	13.70	14.37
21	G*_N7_·C*(w_H_)	16.43	17.33
22	G^*t^·C*_O2_(w_H_)	17.07	17.80
23	G*_N7_·C*(w_H_)	30.33	30.90
24	${\text{G}}_{\text{N}7}^{*\text{t}}\cdot$C*(w_H_)	34.30	35.29
**rHrw**_**H**_			
25	G^*t^·C*(rH)	0.00	0.00
26	G^*t^·C(rw_H_)	0.26	0.88
27	G*_N7_·C*(rw_H_)	10.81	11.72
28	G^*t^·C*_O2_(rw_H_)	18.29	19.49
29	${\text{G}}_{\text{N}7}^{*\text{t}}\cdot$C*(rw_H_)	34.06	34.95

a *Relative Gibbs free energy (T=298.15 K), kcal·mol^−1^*.

b *Relative electronic energy, kcal·mol^−1^*.

Before providing the discussion of the investigated material, let us firstly give attention to the novel mechanisms of the G^*^·C^*^(rWC) tautomerization, which complement the results of the previous work (Brovarets' et al., 2019a).

1. So, in the G^*^·C^*^(rWC) base pair, the non-usual DPT-tautomerization was fixed by the participation of the protons at the N3(C) and N2(G) atoms (Figure 1, part I): G^*^·C^*^(rWC)↔${\text{G}}_{\text{N}2}^{*\text{t}}\cdot$C(rWC). This process is unusual, since the transfer of the proton from the C^*^ to the G^*^ base along the intermolecular (C)N3H.N1(G) H-bond provokes the rotation of the amino group of the G base into the *trans-*position relative to the C2=N3 double bond. As a result, a significantly non-planar ${\text{TS}}_{{\text{G}}^{*}\cdot {\text{C}}^{*}\left(\text{rWC}\right)↔{\text{G}}_{\text{N}2}^{*\text{t}}\cdot \text{C}\left(\text{rWC}\right)}$ of the tautomerization reaction is formed, which proceeds through the asynchronous mechanism and the significantly non-planar product of the tautomerization—the ${\text{G}}_{\text{N}2}^{*\text{t}}\cdot$C(rWC) base pair, which is stabilized by the three intermolecular H-bonds (G)O6H…O2(C), (G)N1H…N3(C), and (C)N4H…N2(G). Its characteristic structural specificity has significant non-planarity and out-of-plane deformation of the purine ring of the O6H, N1H, and N2H atomic groups with *trans-*orientation relatively to the neighboring C2N3 bond.
2. Further, it was found out that Löwdin's G^*^·C^*^(WC) DNA base pair, which is formed from the classic G·C(WC) DNA base pair through the DPT and is stabilized by the participation of the three intermolecular (G)O6H…N4(C), (C)N3H…N1(G), and (G)N2H…O2(C) H-bonds (Brovarets' and Hovorun, 2014a), does not tautomerize in the wobble manner.

In this case all localized transition states of tautomerization in this manner and its pathways are the same as in the case of the wobble mutagenic tautomerization of the G·C(WC) DNA base pair, which has been investigated and described earlier (Brovarets' and Hovorun, 2015a). In other words, in order to tautomerize in the wobble-manner, the Löwdin's G^*^·C^*^(WC) DNA base pair should revert back to the classic G·C(WC) configuration (form) (Brovarets' and Hovorun, 2015a, 2018).

This bright fact allows us to claim that the functional role of the tautomeric G·C(WC) → G^*^·C^*^(WC) transition consists in the removal of the steric obstacles for the conformational G·C(WC) → G^*^·C^*^(rWC) transition (Brovarets' et al., 2019a) and is not directly related to the origin of the spontaneous point mutations—transitions and transversions, as it was suggested earlier (please, refer to work (Brovarets' and Hovorun, 2014a) and references provided therein for more details).

This aforementioned conformational transition, in its turn, guarantees the integration of the G·C(WC) nucleobase pair into the DNA/RNA with parallel strands.

3. Opposite to the previously considered methods both the so-called correct and incorrect DNA base pairs (Brovarets' and Hovorun, 2009, 2015a,b,c,d,e,f; Brovarets' and Hovorun, 2018), the process of the tautomeric wobblization in the investigated G^*^·C^*^(rWC) (Figure 1, parts II and III), and G^*t^·C^*^(H) (Figure 1, part X) base pairs is preceded by the process of the tautomerization *via* the single proton transfer (SPT). At this point, both processes of the wobblization of the G^*^·C^*^(rWC) base pair occur through the joint intermediate—tight G^+^·C^−^ ion pair, which is stabilized by the participation of the three (G)O6^+^H…O2^−^(C), (G)N1^+^H…N3^−^(C), and (G)N2^+^H…N4^−^H(C) H-bonds (Brovarets' and Hovorun, 2014a). This dynamically non-stable intermediate is associated with the local minimum on potential (electronic) energy surface (PES). This situation is observed for the first time. Up until now the commonly accepted idea, that mutagenic tautomerization of the classic DNA base pairs is assisted by the intermediate corresponding to the local minimum on the PES, has not been confirmed.

The first process of the tautomeric wobblization of the G^*^·C^*^(rWC) base pair (Figure 1, part II)—G^*^·C^*^(rWC)↔G·${\text{C}}_{\text{O}2}^{*}$(rWC)↔G·${\text{C}}^{*}\left({\text{rw}}_{\text{WC}}\right)↔{\text{G}}^{*}\cdot$C(rw_WC_)—is *most likely tightly connected with the incorporation of the G*·*C(WC) base pair into the DNA/RNA with parallel strands* (Watson and Crick, 1953b).

Another tautomerization process (Figure 1, part III)—G^*^·C^*^(rWC)↔${\text{G}}_{\text{N}2}^{*}\cdot {\text{C}}^{*}\left({\text{rw}}_{\text{wc}}\right)↔$${\text{G}}^{*}\cdot {\text{C}}_{\text{O}2}^{*}\left({\text{rw}}_{\text{wc}}\right)$, which proceeds through the unique ${\text{TS}}_{\text{G}+\cdot \text{C}-\left(\text{rWC}\right)↔{\text{G}}^{*}\cdot {\text{C}}^{*}\text{O}2\left(\text{rwwc}\right)}$ path with the (G)N1-H-O2(C) covalent bridge, is most probably concerned with the mechanisms of maintaining the RNA spatial architecture due to the incorporation of the non-stable (in the main tautomeric state) Levitt base pair (Crick and Watson, 1954; Levitt, 1969). This suggestion is based on the established structural mechanism of the tautomeric interconversion of the G^*^·C^*^(rWC) pair into the eight *stable planar tautomeric forms of the Levitt base pair* (Watson and Crick, 1953a) (Figure 1, parts IV-VI)—${\text{G}}^{*\text{t}}\cdot {{\text{C}}^{*}}_{\text{O}2}\left({\text{rw}}_{\text{WC}}\right)$, ${\text{G}}^{*}\cdot {\text{C}}_{\text{O}2}^{*}\left({\text{rw}}_{\text{WC}}\right)$, ${\text{G}}^{*\text{t}}\cdot {\text{C}}_{\text{O}2}^{*\text{t}}\left({\text{rw}}_{\text{WC}}\right)$, ${\text{G}}^{*}\cdot {\text{C}}_{\text{O}2}^{*\text{t}}\left({\text{rw}}_{\text{WC}}\right)$, ${\text{G}}_{\text{N}2}^{*}\cdot {\text{C}}^{*}\left({\text{rw}}_{\text{WC}}\right)$, ${\text{G}}_{\text{N}2}^{*\text{t}}\cdot {\text{C}}^{*}\left({\text{rw}}_{\text{WC}}\right)$, ${\text{G}}_{\text{N}2}^{*}\cdot {\text{C}}^{*\text{t}}\left({\text{rw}}_{\text{WC}}\right)$, and ${\text{G}}_{\text{N}2}^{*\text{t}}\cdot {\text{C}}^{*\text{t}}\left({\text{rw}}_{\text{WC}}\right)$ (Figure 2, Table 1)—and in principle, allows us to understand the dynamic of the formation of the Levitt base pair, which has not been considered before in the literature. It would be interesting to investigate how the tautomers of the Levitt base pair is stabilized in RNA by the H-bonds and surrounding environment further in the future (Oliva et al., 2007).

4. A quite interesting situation is observed for the tautomeric wobblization of the G^*t^·C^*^(H) base pair (Figure 1, part VII): G^*t^·C^*^(H)↔${\text{G}}_{\text{N}7}^{*}\cdot$C(C)↔G^*t^·${\text{C}}_{\text{O}2}^{*}$(w_H_)↔${\text{C}}_{\text{N}7}^{*}\cdot$C^*^(w_H_).

The transition of the ${\text{C}}_{\text{O}2}^{*}$ tautomer of the cytosine (C) within the ${\text{C}}^{*\text{t}}\cdot {\text{C}}_{\text{O}2}^{*}$(w_H_) base pairs with *cis*-orientation of the N4H C-imino group into the *trans*-orientation through its inversion leads to the decreasing of the energy in the tautomerization (Figure 1, part VIII): G^*t^· ${\text{C}}_{\text{O}2}^{*}$(w_H_)↔G^*t^·${\text{C}}_{\text{O}2}^{*\text{t}}\left({\text{w}}_{\text{H}}\right)↔$${\text{G}}_{\text{N}7}^{*}\cdot$${\text{C}}^{*\text{t}}\left({\text{w}}_{\text{H}}\right)↔$${\text{G}}_{\text{N}7}^{*}\cdot$${\text{C}}^{*}\left({\text{w}}_{\text{H}}\right)$. This decreasing of energy occurs when the affinity of the ${\text{C}}_{\text{O}2}^{*\text{t}}$ tautomer according to the “complementary” G^*t^ tautomer is higher than the ${\text{C}}_{\text{O}2}^{*}$ tautomer. This decreasing of the energy with excess overrides the increasing of the internal energy of the ${\text{C}}_{\text{O}2}^{*}$ tautomer at its tautomerization ${\text{C}}_{\text{O}2}^{*}\to$${\text{C}}_{\text{O}2}^{*\text{t}}$.

In the another pathway of the tautomeric wobblization of the G^*t^·C^*^(H) base pair (Figure 1, part VII, VIII) the decreasing of energy in the course of the process is achieved by the conformational transition of the G^*t^ tautomer within the ${\text{G}}^{*\text{t}}\cdot \text{C}\left({\text{w}}_{\text{H}}\right)$ complex into the low-energy mutagenic tautomeric form ${\text{G}}_{\text{N}7}^{*}$, which is zwitterion.

5. At this, the G^*t^·C(w_H_)↔${\text{G}}_{\text{N}7}^{*\text{t}}\cdot$C^*^(w_H_) DPT tautomerization process does not really occur, since its barrier is negative under normal conditions (Figure 1, part IX): G^*t^·C(w_H_)↔G^*^·C(w_H_)↔${\text{G}}_{\text{N}7}^{*}\cdot$C^*^(w_H_)↔${\text{G}}_{\text{N}7}^{*\text{t}}\cdot$C^*^(w_H_). The same situation is also observed for the G^*t^·C^*^(H)↔${\text{G}}_{\text{N}7}^{*}\cdot$C(H)↔G^*t^·C(w_H_)↔${\text{G}}_{\text{N}7}^{*\text{t}}\cdot$C^*^(w_H_) DPT tautomerization (Figure 1, part X).
6. Tautomeric wobblization of the G^*t^·C^*^(rH) base pair (Figure 1, part XI) occurs through the two traditional pathways without any preparatory SPT stages through the TSs, which represent themselves as the covalently bonded tight G^+^·C^−^ ion pairs in reverse Hoogsteen conformation, which are only supported by two H-bonds: G^*t^·C^*^(rH)↔G^*t^·${\text{C}}_{\text{O}2}^{*}$(rw_H_)↔${\text{G}}_{\text{N}7}^{*\text{t}}\cdot$C^*^(rw_H_) and G^*t^·C^*^(rH)↔G^*t^·C^*^(rw_H_)↔${\text{G}}_{\text{N}7}^{*}\cdot$C^*^(rw_H_). The transition of the G^*t^ tautomer within the G^*t^·${\text{C}}_{\text{O}2}^{*}$(rw_H_) complex into the G^*^ mutagenic tautomer through the orthogonal TS decreases the energy of the further process of tautomerization.
7. Also, in addition to the previously revealed processes, DPT tautomerization was also fixed by the participation of the proton at the C8 carbon atom of G, which lead to the dynamically-stable, but short-lived, complexes by the participation of the yilidic forms of the G base (Figure 1, parts IX-XI).
8. Finally, there are three more fixed mysteries, which deserve more attention. Several G·C base pairs, in which both bases were in the rare tautomeric form and their energy of stabilization significantly exceeded the analogical values for the classic G·C(WC) DNA base pair were fixed.

Despite the structural softness of the heterocycles of the G and C bases for the out-of-plane deformational bending (Hovorun et al., 1999), it was not revealed that there was any deviation from the plane in the investigated processes of the tautomerization of the base pairs.

Obtained data convincingly show that among all possible tautomeric wobblizations of the G^*^·C^*^(rWC), G^*t^·C^*^(H), and G^*t^·C^*^(rH) DNA base pairs, which possess Watson-Crick, Hoogsteen, and reverse Hoogsteen configurations and both monomers of which are in the rare tautomeric form, at least one non-dissociative transition was absent, which would recover the tautomeric status of both the G^*^/G^*t^ and C^*^ bases to the canonical G and C bases, correspondingly. This fact altogether with the results, obtained in our previous work (Brovarets' et al., 2019a), soundly exhibits why the Watson-Crick DNA base pairs were chosen for the building of genetic material (Brovarets' et al., 2018a).

## Conclusion

Concluding the obtained results, we arrived to a summation after providing an investigation of the tautomeric wobblization of the biologically-important G·C(WC), G^*^·C^*^(WC), G^*^·C^*^(rWC), G^*t^·C^*^(H), and G^*t^·C^*^(rH) nucleobase pairs and extended the existing thoughts about the microstructural mechanisms of these processes, as well as about their functional roles. Thus, it was established that the G·C base pair is the most likely to be incorporated into the DNA/RNA double helix with parallel strands in the form of the G^*^·${\text{C}}_{\text{O}2}^{*}$(rWC), G·C^*^(rw_WC_), and G^*^·C(rw_WC_) tautomers, which are in rapid tautomeric equilibrium with each other.

For the first time we have formulated rules, defining these biologically-important processes.

## Data Availability Statement

All datasets generated for this study are included in the article/supplementary material.

## Author Contributions

OB: idea formulation, setting of the task, calculation of the data, building of the graphs, data extrapolation, preparing, and proofreading of the draft of the manuscript. AM: idea formulation, calculation of the data, building of the graphs, preparing, and proofreading of the draft of the manuscript. DH: idea formulation, preparing, and proofreading of the draft of the manuscript. All authors contributed to the article and approved the submitted version.

## Conflict of Interest

The authors declare that the research was conducted in the absence of any commercial or financial relationships that could be construed as a potential conflict of interest.

## References

[B1] Alvey H. S. Gottardo F. L. Nikolova E. N. Al-Hashimi H. M. (2014). Widespread transient Hoogsteen base-pairs in canonical duplex DNA with variable energetics. Nat. Comm. 5, 4786–4794. 10.1038/ncomms5786 25185517 PMC4537320

[B2] Atkins P. W. (1998). Physical Chemistry. Oxford: Oxford University Press.

[B3] Bader R. F. W. (1990). Atoms in Molecules: A Quantum Theory. Oxford: Oxford University Press.

[B4] Bayley S. T. (1951). The dielectric properties of various solid crystalline proteins, amino acids and peptides. Trans. Faraday Soc. 47, 509–517. 10.1039/tf9514700509

[B5] Boys S. F. Bernardi F. (1970). The calculation of small molecular interactions by the differences of separate total energies. Some procedures with reduced errors. Mol. Phys. 19, 553–566. 10.1080/00268977000101561

[B6] Brovarets' O. O. Hovorun D. M. (2009). Physicochemical mechanism of the wobble DNA base pairs Gua·Thy and Ade·Cyt transition into the mismatched base pairs Gua^*^·Thy and Ade·Cyt^*^ formed by the mutagenic tautomers. Ukr. Bioorg. Acta 8, 12–18.

[B7] Brovarets' O. O. Hovorun D. M. (2010a). Quantum-chemical investigation of tautomerization ways of Watson-Crick DNA base pair guanine-cytosine. Ukr. Biochem. J. 82, 55–60. 21328878

[B8] Brovarets' O. O. Hovorun D. M. (2010b). Quantum-chemical investigation of the elementary molecular mechanisms of pyrimidine·purine transversions. Ukr. Biochem. J. 82, 57–67. 21674962

[B9] Brovarets' O. O. Hovorun D. M. (2014a). Why the tautomerization of the G·C Watson–Crick base pair *via* the DPT does not cause point mutations during DNA replication? QM and QTAIM comprehensive analysis. J. Biomol. Struct. Dynam. 32, 1474–1499. 10.1080/07391102.2013.822829 23909623

[B10] Brovarets' O. O. Hovorun D. M. (2014b). Can tautomerisation of the A·T Watson-Crick base pair *via* double proton transfer provoke point mutations during DNA replication? A comprehensive QM and QTAIM analysis. J. Biomol. Struct. Dynam. 32, 127–154. 10.1080/07391102.2012.755795 23383960

[B11] Brovarets' O. O. Hovorun D. M. (2015a). New structural hypostases of the A·T and G·C Watson-Crick DNA base pairs caused by their mutagenic tautomerisation in a wobble manner: a QM/QTAIM prediction. RSC Adv. 5, 99594–99605. 10.1039/C5RA19971A

[B12] Brovarets' O. O. Hovorun D. M. (2015b). Tautomeric transition between wobble A·C DNA base mispair and Watson-Crick-like A·C^*^ mismatch: microstructural mechanism and biological significance. Phys. Chem. Chem. Phys. 17, 15103–15110. 10.1039/C5CP01568E 25994250

[B13] Brovarets' O. O. Hovorun D. M. (2015c). How many tautomerisation pathways connect Watson-Crick-like G^*^·T DNA base mispair and wobble mismatches? J. Biomol. Struct. Dynam. 33, 2297–2315. 10.1080/07391102.2015.1046936 25932960

[B14] Brovarets' O. O. Hovorun D. M. (2015d). Wobble↔Watson-Crick tautomeric transitions in the homo-purine DNA mismatches: a key to the intimate mechanisms of the spontaneous transversions. J. Biomol. Struct. Dynam. 33, 2710–2715. 10.1080/07391102.2015.1077737 26237090

[B15] Brovarets' O. O. Hovorun D. M. (2015e). Novel physico-chemical mechanism of the mutagenic tautomerisation of the Watson–Crick-like A·G and C·T DNA base mispairs: a quantum-chemical picture. RSC Adv. 5, 66318–66333. 10.1039/C5RA11773A

[B16] Brovarets' O. O. Hovorun D. M. (2015f). A novel conception for spontaneous transversions caused by homo-pyrimidine DNA mismatches: a QM/QTAIM highlight. Phys. Chem. Chem. Phys. 17, 21381–21388. 10.1039/C5CP03211C 26219928

[B17] Brovarets' O. O. Hovorun D. M. (2015g). The nature of the transition mismatches with Watson-Crick architecture: the G^*^·T or G·T^*^ DNA base mispair or both? A QM/QTAIM perspective for the biological problem. J. Biomol. Struct. Dynam. 33, 925–945. 10.1080/07391102.2014.924879 24842163

[B18] Brovarets' O. O. Hovorun D. M. (2018). “Renaissance of the tautomeric hypothesis of the spontaneous point mutations in DNA: new ideas and computational approaches,” in Mitochondrial DNA—New Insights, ed Herve Seligmann (London: IntechOpen), 31–55. 10.5772/intechopen.77366

[B19] Brovarets' O. O. Oliynyk T. A. Hovorun D. M. (2019a). Novel tautomerisation mechanisms of the biologically important conformers of the reverse Löwdin, Hoogsteen, and reverse Hoogsteen G^*^·C^*^ DNA base pairs *via* proton transfer: a quantum-mechanical survey. Front. Chem. 7:597. 10.3389/fchem.2019.00597 31620420 PMC6759773

[B20] Brovarets' O. O. Oliynyk T. A. Hovorun D. M. (2019b). “Novel conformers of the G·C DNA base pair and their mutual interconversions *via* the proton transfer: a quantum-mechanical study” in Joint 12^th^ EBSA Congress and 10^th^ ICBP-IUPAP Congress, Vol. 48 (Madrid), S90. 10.1007/s00249-019-01373-4 31620420 PMC6759773

[B21] Brovarets' O. O. Tsiupa K. S. Dinets A. Hovorun D. M. (2018c). Unexpected routes of the mutagenic tautomerization of the T nucleobase in the classical A·T DNA base pairs: A QM/QTAIM comprehensive view. Front. Chem. 6:532. 10.3389/fchem.2018.00532 30538979 PMC6277528

[B22] Brovarets' O. O. Tsiupa K. S. Hovorun D. M. (2018a). The A·T(rWC)/A·T(H)/A·T(rH) ↔ A·T^*^(rw_WC_)/A·T^*^(w_H_)/A·T^*^(rw_H_) mutagenic tautomerization *via* sequential proton transfer: a QM/QTAIM study. RSC Adv. 8, 13433–13445. 10.1039/C8RA01446A PMC9079753 35542561

[B23] Brovarets' O. O. Tsiupa K. S. Hovorun D. M. (2018b). Unexpected A·T(WC)↔A·T(rWC)/A·T(rH) and A·T(H)↔A·T(rH)/A·T(rWC) conformational transitions between the classical A·T DNA base pairs: A QM/QTAIM comprehensive study. Int. J. Quantum. Chem. 118:e25674 10.1002/qua.25692

[B24] Brovarets' O. O. Tsiupa K. S. Hovorun D. M. (2018d). Non-dissociative structural transitions of the Watson-Crick and reverse Watson-Crick A·T DNA base pairs into the Hoogsteen and reverse Hoogsteen forms. Sci. Repts. 8:10371. 10.1038/s41598-018-28636-y 29991693 PMC6039495

[B25] Brovarets' O. O. Tsiupa K. S. Hovorun D. M. (2018e). Novel pathway for mutagenic tautomerization of classical A·T DNA base pairs *via* sequential proton transfer through quasi-orthogonal transition states: a QM/QTAIM investigation. PLoS ONE 13:e0199044. 10.1371/journal.pone.0199044 29949602 PMC6021055

[B26] Brovarets' O. O. Tsiupa K. S. Hovorun D. M. (2018f). Surprising conformers of the biologically important A·T DNA base pairs: QM/QTAIM proofs. Front. Chem. 6:8. 10.3389/fchem.2018.00008 29536003 PMC5835050

[B27] Brovarets' O. O. Zhurakivsky R. O. Hovorun D. M. (2014). Does the tautomeric status of the adenine bases change upon the dissociation of the A^*^·A_syn_ Topal-Fresco DNA mismatch? A combined QM and QTAIM atomistic insight. Phys. Chem. Chem. Phys. 16, 3715–3725. 10.1039/c3cp54708f 24418908

[B28] Brovarets' O. O. Zhurakivsky R. O. Hovorun D. M. (2015). DPT tautomerisation of the wobble guanine·thymine DNA base mispair is not mutagenic: QM and QTAIM arguments. J. Biomol. Struct. Dynam. 33, 674–689. 10.1080/07391102.2014.897259 24650179

[B29] Crick F. H. C. Watson J. D. (1954). The complementary structure of deoxyribonucleic acid. Proc. Roy. Soc. A 223, 80–96. 10.1098/rspa.1954.0101

[B30] Cukrowski I. Matta C. F. (2010). Hydrogen–hydrogen bonding: a stabilizing interaction in strained chelating rings of metal complexes in aqueous phase. Chem. Phys. Lett. 499, 66–69. 10.1016/j.cplett.2010.09.013

[B31] Dewar M. J. S. Storch D. M. (1985). Alternative view of enzyme reactions. Proc. Natl. Acad. Sci. U. S. A. 82, 2225–2229. 10.1073/pnas.82.8.2225 3857576 PMC397529

[B32] El-Sayed A. A. Tamara Molina A. Alvarez-Ros M. C. Alcolea Palafox M. (2015). Conformational analysis of the anti-HIV Nikavir prodrug: comparisons with AZT and thymidine, and establishment of structure-activity relationships/tendencies in other 6'-derivatives. J. Biomol. Struct. Dynam. 33, 723–748. 10.1080/07391102.2014.909743 24762127

[B33] Florian J. Hrouda V. Hobza P. (1994). Proton transfer in the adenine-thymine base pair. J. Am. Chem. Soc. 116,1457–1460. 10.1021/ja00083a03422414119

[B34] Frisch M. J. Head-Gordon M. Pople J. A. (1990). Semi-direct algorithms for the MP2 energy and gradient. Chem. Phys. Lett. 166, 281–289. 10.1016/0009-2614(90)80030-H

[B35] Frisch M. J. Trucks G. W. Schlegel H. B. Scuseria G. E. Robb M. A. Cheeseman J. R. (2010). GAUSSIAN 09 (Revision B.01). Wallingford CT: Gaussian Inc.

[B36] García-Moreno B. E. Dwyer J. J. Gittis A. G. Lattman E. E. Spencer D. S. Stites W. E. (1997). Experimental measurement of the effective dielectric in the hydrophobic core of a protein. Biophys. Chem. 64, 211–224. 10.1016/S0301-4622(96)02238-7 9127946

[B37] Godbeer A. D. Al-Khalili J. S. Stevenson P. D. (2015). Modelling proton tunnelling in the adenine-thymine base pair. Phys. Chem. Chem. Phys. 7, 13034–13044. 10.1039/C5CP00472A 25913695

[B38] Gorb L. Podolyan Y. Dziekonski P. Sokalski W. A. Leszczynski J. (2004). Double-proton transfer in adenine–thymine and guanine–cytosine base pairs. A post Hartree–Fock *ab initio* study. J. Am. Chem. Soc. 126, 10119–10129. 10.1021/ja049155n 15303888

[B39] Gutowski M. Van Lenthe J. H. Verbeek J. Van Duijneveldt F. B. Chalasinski G. (1986). The basis set superposition error in correlated electronic structure calculations. Chem. Phys. Lett. 124, 370–375. 10.1016/0009-2614(86)85036-9

[B40] Hariharan P. C. Pople J. A. (1973). The influence of polarization functions on molecular orbital hydrogenation energies. Theor. Chim. Acta 28, 213–222. 10.1007/BF00533485

[B41] Hovorun D. M. Gorb L. Leszczynski J. (1999). From the nonplanarity of the amino group to the structural nonrigidity of the molecule: a post-Hartree-Fock *ab initio* study of 2-aminoimidazole. Int. J. Quantum. Chem. 75, 245–253. 10.1002/(SICI)1097-461X(1999)75:3<245::AID-QUA14>3.0.CO;2-0

[B42] Keith T. A. (2010). AIMAll (Version 10.07.01). Available online at: aim.tkgristmill.com (accessed October 23, 2020).

[B43] Kendall R. A. Dunning Jr T. H. Harrison R.J. (1992). Electron affinities of the first-row atoms revisited. Systematic basis sets and wave functions. J. Chem. Phys. 96, 6796–6806. 10.1063/1.462569

[B44] Krishnan R. Binkley J. S. Seeger R. Pople J. A. (1980). Self-consistent molecular orbital methods. XX. A basis set for correlated wave functions. J. Chem. Phys. 72, 650–654. 10.1063/1.438955

[B45] Lecomte C. Espinosa E. Matta C. F. (2015). On atom–atom “short contact” bonding interactions in crystals. IUCrJ 2, 161–163. 10.1107/S2052252515002067 25866651 PMC4392409

[B46] Lee C. Yang W. Parr R. G. (1988). Development of the Colle-Salvetti correlation-energy formula into a functional of the electron density. Phys. Rev. B. 37, 785–789. 10.1103/PhysRevB.37.785 9944570

[B47] Levitt M. (1969). Detailed molecular model for transfer ribonucleic acid. Nature 224, 759–763. 10.1038/224759a0 5361649

[B48] Löwdin P.-O. (1963). Proton tunneling in DNA and its biological implications. Rev. Mod. Phys. 35, 724–732. 10.1103/RevModPhys.35.724

[B49] Löwdin P.-O. (1966). “Quantum genetics and the aperiodic solid: some aspects on the biological problems of heredity, mutations, aging, and tumors in view of the quantum theory of the DNA molecule,” in Advances in Quantum Chemistry, ed Löwdin P.-O. (New York, NY; London: Academic Press), 213–360. 10.1016/S0065-3276(08)60076-3

[B50] Matta C. F. (2010). How dependent are molecular and atomic properties on the electronic structure method? Comparison of Hartree-Fock, DFT, and MP2 on a biologically relevant set of molecules. J. Comput. Chem. 31, 1297–1311. 10.1002/jcc.21417 19882732

[B51] Matta C. F. (2014). Modeling biophysical and biological properties from the characteristics of the molecular electron density, electron localization and delocalization matrices, and the electrostatic potential. J. Comput. Chem. 35, 1165–1198. 10.1002/jcc.23608 24777743 PMC4368384

[B52] Matta C. F. Castillo N. Boyd R. J. (2006). Atomic contributions to bond dissociation energies in aliphatic hydrocarbons. J. Chem. Phys. 125:204103. 10.1063/1.2378720 17144686

[B53] Matta C. F. Hernández-Trujillo J. (2003). Bonding in polycyclic aromatic hydrocarbons in terms of the electron density and of electron delocalization. J. Phys. Chem. A 107, 7496–7504. 10.1021/jp034952d

[B54] Mertz E. L. Krishtalik L. I. (2000). Low dielectric response in enzyme active site. Proc. Natl. Acad. Sci. U. S. A. 97, 2081–2086. 10.1073/pnas.050316997 10681440 PMC15757

[B55] Nikolova E. N. Zhou H. Gottardo F. L. Alvey H. S. Kimsey I. J. Al-Hashimi H. M. (2014). A historical account of Hoogsteen base pairs in duplex DNA. Biopolymers 99, 955–968. 10.1002/bip.22334 23818176 PMC3844552

[B56] Oliva R. Tramontano A. Cavallo L. (2007). Mg^2+^ binding and archaeosine modification stabilize the G15–C48 Levitt base pair in tRNAs. RNA 13, 1427–1436. 10.1261/rna.574407 17652139 PMC1950755

[B57] Palafox M. A. (2014). Molecular structure differences between the antiviral nucleoside analogue 5-iodo-2‘-deoxyuridine and the natural nucleoside 2‘-deoxythymidine using MP2 and DFT methods: conformational analysis, crystal simulations, DNA pairs and possible behavior. J. Biomol. Struct. Dynam. 32, 831–851. 10.1080/07391102.2013.789402 23731482

[B58] Parr R. G. Yang W. (1989). Density-Functional Theory of Atoms and Molecules. Oxford: Oxford University Press.

[B59] Peng C. Ayala P. Y. Schlegel H. B. Frisch M. J. (1996). Using redundant internal coordinates to optimize equilibrium geometries and transition states. J. Comput. Chem. 17, 49–56. 10.1002/(SICI)1096-987X(19960115)17:1<49::AID-JCC5>3.0.CO;2-0

[B60] Petrushka J. Sowers L. C. Goodman M. (1986). Comparison of nucleotide interactions in water, proteins, and vacuum: model for DNA polymerase fidelity. Proc. Natl. Acad. Sci. U. S. A. 83, 1559–1562. 10.1073/pnas.83.6.1559 3456600 PMC323122

[B61] Poltev V. I. Anisimov V. M. Sanchez C. Deriabina A. Gonzalez E. Garcia D.. (2016). Analysis of the conformational features of Watson–Crick duplex fragments by molecular mechanics and quantum mechanics methods. Biophysics 61, 217–226. 10.1134/S0006350916020160 27192827

[B62] Pous J. Urpi L. Subirana J. A. Gouyette C. Navaza J. Campos J. L. (2008). Stabilization by extra-helical thymines of a DNA duplex with Hoogsteen base pairs. J. Am. Chem. Soc. 130, 6755–6760. 10.1021/ja078022+ 18447354

[B63] Saenger W. (1984). Principles of Nucleic Acid Structure. New York, NY: Springer 10.1007/978-1-4612-5190-3

[B64] Sordo J. A. (2001). On the use of the Boys–Bernardi function counterpoise procedure to correct barrier heights for basis set superposition error. J. Mol. Struct. 537, 245–251. 10.1016/S0166-1280(00)00681-3

[B65] Sordo J. A. Chin S. Sordo T. L. (1988). On the counterpoise correction for the basis set superposition error in large systems. Theor. Chim. Acta 74, 101–110. 10.1007/BF00528320

[B66] Srivastava R. (2019). The role of proton transfer on mutations. Front Chem. 7:536. 10.3389/fchem.2019.00536 31497591 PMC6712085

[B67] Szabat M. Kierzek R. (2017). Parallel-stranded DNA and RNA duplexes: structural features and potential applications. FEBS J. 284, 3986–3998. 10.1111/febs.14187 28771935

[B68] Tirado-Rives J. Jorgensen W. L. (2008). Performance of B3LYP density functional methods for a large set of organic molecules. J. Chem. Theory Comput. 4, 297–306. 10.1021/ct700248k 26620661

[B69] Topal M. D. Fresco J. R. (1976). Complementary base pairing and the origin of substitution mutations. Nature 263, 285–289. 10.1038/263285a0 958482

[B70] Turaeva N. Brown-Kennerly V. (2015). Marcus model of spontaneous point mutation in DNA. Chem. Phys. 461, 106–110. 10.1016/j.chemphys.2015.09.005

[B71] Watson J. D. Crick F. H. C. (1953a). Molecular structure of nucleic acids: a structure for deoxyribose nucleic acid. Nature 171, 737–738. 10.1038/171737a0 13054692

[B72] Watson J. D. Crick F. H. C. (1953b). The structure of DNA. Cold. Spring Harb. Symp. Quant. Biol. 18, 123–131. 10.1101/SQB.1953.018.01.020 13168976

